# Intracytoplasmic sperm injection in sturgeon species: A promising reproductive technology of selected genitors

**DOI:** 10.3389/fvets.2022.1054345

**Published:** 2022-12-23

**Authors:** Effrosyni Fatira, Miloš Havelka, Taiju Saito, José Landeira, Marek Rodina, David Gela, Martin Pšenička

**Affiliations:** ^1^Research Institute of Fish Culture and Hydrobiology, South Bohemian Research Center of Aquaculture and Biodiversity of Hydrocenoses, University of South Bohemia Ceske Budejovice, Ceské Budějovice, Czechia; ^2^Instituto de Oceanografía y Cambio Global, IOCAG, Universidad de Las Palmas de Gran Canaria, Las Palmas de Gran Canaria, Spain; ^3^Nishiura Station, South Ehime Fisheries Research Center, Ehime University, Matsuyama, Japan

**Keywords:** assisted reproduction, intracytoplasmic sperm injection, sturgeon, embryo, larva

## Abstract

Sturgeons are the most endangered species group and their wild populations continue to decrease. In this study, we apply intracytoplasmic sperm injection (ICSI), an assisted reproductive technology, for the first time in endangered and critically endangered sturgeons. Using various egg-sperm species combinations we performed different ICSI experiments with immobilized pre- or non-activated spermatozoa, single or many, fresh or cryopreserved. Then we evaluated the fertilization success as well as the paternity of the resultant embryos and larvae. Surprisingly, all experimental groups exhibited embryonic development. Normal-shaped feeding larvae produced in all egg-sperm species-combination groups after ICSI using single fresh-stripped non-activated spermatozoa, in one group after ICSI using single fresh-stripped pre-activated spermatozoa, and in one group after ICSI using multiple fresh-stripped spermatozoa. ICSI with single cryopreserved non-activated spermatozoa produced neurula stage embryos. Molecular analysis showed genome integration of both egg- and sperm-donor species in most of the ICSI transplants. Overall, ICSI technology could be used as an assisted reproduction technique for producing sturgeons to rescue valuable paternal genomes.

## 1. Introduction

Intracytoplasmic sperm injection (ICSI) is an assisted reproductive technology that aims to treat male factor infertility in both humans and animals as well as to make a significant contribution to basic science as a useful tool in the research of gamete biology (1–7). The technology involves the microinjection of a single sperm or a sperm head (nucleus) into the cytoplasm of a mature metaphase II egg, by passing the natural process of egg-sperm interaction (8). Although the cell membranes of the egg and sperm are not in contact, sperm delivered in this manner initiates Ca^2+^ responses in the egg that closely resemble those initiated by *in vitro* fertilization (9–12). Research in mice revealed that the ICSI technology produced viable fetuses that did not exhibit more spontaneous mutations than naturally conceived and gestated fetuses (13). The ICSI technology has been demonstrated in humans to support high rates of development to term (14, 15).

The use of ICSI technology provides the possibility to fertilize eggs with semen on reduced sperm concentration or motility (16). In this sense, ICSI could be an option to achieve *in vitro*-produced embryos in species like camelids in which the recovery of adequate quantity and quality of sperm for conventional *in vitro* fertilization is problematic (17). In addition, the ICSI technology offers fertilization using sexed semen (18), cryopreserved semen (19, 20), lyophilized semen (21, 22), or semen collected postmortem (23). The last provides the possibility to produce offspring from gametes of deceased animals (23). In this sense, it may be a useful reproductive technology for endangered species when no motile or reduced motility sperm is retrieved from cadavers (24). Furthermore, ICSI could contribute as a useful tool in conservation efforts of endangered species in which the males might develop a higher proportion of abnormal sperm during captivity. These are the cases for the clouded leopard and cheetah, where a high level of sperm abnormalities has been detected in their captive populations (25, 26). ICSI technology could have a useful application in aquaculture because it provides the possibility of hybridization between homogeneous and even heterogeneous fish species, but care should be taken to prevent genetic pollution to natural populations.

ICSI was first performed in starfish (27), followed by sea urchin (28), mammals such as human (14, 29), farm animals like sheep (5), horse (3), and pig (4), as well as amphibians like *Xenopus* (30, 31), with various successes. More recently, after interspecific ICSI using domestic cat eggs and wild felids such as cheetah or leopard fresh-stripped spermatozoa, developmental rates until blastocyst have been achieved (32). Success until blastocyst was also reported in an Italian endangered sheep breed named Pagliarola when lyophilised spermatozoa was used (22).

ICSI in fish has been performed in a few species like the crucian carp (33), zebrafish (34), Nile tilapia (19), medaka (7), and dojo loach (35). Hatching rates using fresh-stripped spermatozoa have been succeeded in medaka (13.4%) (7) and in Nile tilapia (8.5 %) (19), while production of fertile adults has been successful only in medaka (5 %) (36). To our knowledge, the ICSI technology has not yet been applied to large endangered fish species. Recent efforts have been made to apply assisted reproductive technologies in sturgeon, the most endangered group of animals (37). Application of the somatic cell nuclear transfer (SCNT) technology in sturgeons (38, 39) yielded a success rate of 0.8% in producing fin cell-donor-derived embryos (39).

Parmar et al. (8) proposed the ICSI technology as the last resort of species conservation when all other conventional methods of insemination fail. In the aim to put more arrows in the quiver for the conservation of endangered sturgeon species, the current study applies for the first time ICSI in sturgeons. We evaluate the ICSI technology using the endangered sterlet, *Acipenser ruthenus*, the critically endangered Siberian sturgeon, *Acipenser baerii*, the critically endangered beluga sturgeon, *Huso huso*, and the critically endangered Russian sturgeon, *Acipenser gueldenstaedtii* (37) in various egg-sperm species combinations. Using non-activated unfertilized eggs arrested in meiosis II stage, we performed different ICSI experiments. To start with, the ICSI of a single non-activated or pre-activated for 15 s fresh-stripped immobilized spermatozoa was used. The later experiment tested the spermatozoa efficiency to fertilize with induced acrosome reaction that showed to have a positive outcome in *in vitro* fertilization (40). Secondly, following the sturgeon reproductive biology, we applied ICSI of multiple non-activated fresh-stripped spermatozoa. Polyspermy happens naturally in the sturgeon populations (41), resulting in viable offspring after *in vitro* fertilization (42). Last but not least, after ICSI of single non-activated cryopreserved spermatozoa of 40 % motility, we tested the possibility to fertilize sturgeon eggs. After the different ICSI experiments, the success of fertilization as well as the paternity of the resulted embryos and larvae have been evaluated.

## 2. Methods

### 2.1. Ethics

The ICSI experiments took place at the aquaculture facility of the Genetic Fisheries Center and Laboratory of Germ Cells at the Faculty of Fisheries and Protection of Waters, University of South Bohemia in Ceské Budějovice, Vodnany, Czech Republic. All experimental procedures were performed in accordance with national (reference number: 2293/2015-MZE-17214) and institutional guidelines on animal experimentation and care and approved by the Animal Research committee of the University of South Bohemia in Ceské Budějovice.

### 2.2. Fish and gamete collection

During the ICSI experiments, a mature female Russian sturgeon (23–24-year-old), Siberian sturgeon (14–15-year-old), and beluga (22–23-year-old), as well as two mature female sterlets (4–5-year-old), were used as egg-donor species. Moreover, a mature male of sterlet (5–6-year-old), Siberian sturgeon (14–15-year-old), and beluga sturgeon (22–23-year-old) were used as sperm-donor species. All fish were kept at constant temperatures of 15°C at least 1 week before collection of gametes. In order to induce ovulation, the females were injected with an intramuscular injection of carp pituitary homogenized extract (CPE) at a dose of 5 mg/kg body weight (b.w.) in two steps: first with 0.5 mg/kg b.w. and second with 4.5 mg/kg b.w. 12 h after the first injection. Egg collection in sterlets was performed 22–24 h after the second injection. Beluga, Siberian, and Russian sturgeon females were stripped 28–30 h after the second injection.

In order to induce spermiation, the males were injected once (4 mg/kg b.w.) with CPE and spermatozoa was collected 24–36 h after hormonal injection by use of a catheter from the urogenital papilla, transferred to a separate cell culture container (250 mL), and stored at 4°C until sampling (1–3 h).

### 2.3. Gametes' preparation

Non-activated and unfertilized eggs from sterlet, Siberian sturgeon, Russian sturgeon, and beluga arrested in meiosis II stage were washed three times for 5 min with Phosphate Buffered Saline (PBS) to remove debris and somatic cells from the spawning (38). Then they were placed in a 6 cm^2^ petri dish filled with PBS at 15°C for ICSI.

To simulate reduced motility spermatozoa with acrosomal reaction retrieved from cadavers, we pre-activated the spermatozoa before ICSI. In an Eppendorf tube (1.5 ml) a drop of fresh stripped spermatozoa (1.5 μl) from sterlet or Siberian sturgeon was diluted (1/50) in filtrated water (15°C) for its activation for 15 s. The pre-activated spermatozoa were then transferred in PBS to return in inertia and held at 4°C until its use for the ICSI experiments.

Spermatozoa cryopreservation from beluga sturgeon was performed according to Glogowski et al. (43). Fresh sperm was diluted with cryomedium in a ratio of 1:1. The composition of cryomedium was 10 % ethanol, 23.4 mM sucrose, 0.25 mM KCl, and 30 mM Tris with pH adjusted to 8.0 by using HCl. The diluted sperm with cryomedium was pipetted into 0.5 ml pejets and kept 3 cm above liquid nitrogen surface for 10 min. Afterwards, the pejets were immersed and stored in liquid nitrogen until use. Beluga sturgeon's spermatozoa thawing was performed in a 36°C water bath for 15 s.

Spermatozoon motility was evaluated visually for the percentage of motile spermatozoa after activation and total duration of motility (in seconds). Motility parameters were measured immediately after initiation of spermatozoon activation until 100 % of spermatozoa were immotile. To induce the initiation of spermatozoon motility, a 49 μl drop of medium was placed on a glass slide and then a drop of 1 μl fresh spermatozoa was diluted using a microsampler. All experiments were performed in triplicate at room temperature (17–20°C), using light microscopy under 400 × magnification. To avoid subjective bias, all measurements were carried out by the same experimenter.

### 2.4. Egg-sperm species combinations, ICSI experiments, treatment, and culture of the embryos

In order to perform ICSI using single spermatozoa, fresh (non- or pre-activated) or cryopreserved, we placed a drop of non-diluted sperm (1.5 μl) close to the eggs. Both eggs and spermatozoa remained inactivated in a petri dish filled with PBS (15°C). To assure that the microinjection needle would aspirate only one spermatozoon at a time, the oil running through the microneedle was creating a small drop that was carefully removed before aspiration. This procedure was used to aspirate as little PBS as possible together with the single spermatozoon. ICSI using fresh single non- or pre- activated spermatozoa was performed between beluga eggs and Siberian sturgeon spermatozoa, between Siberian sturgeon eggs and sterlet spermatozoa, as well as between Russian sturgeon eggs and sterlet spermatozoa. ICSI using cryopreserved single spermatozoa was performed between sterlet eggs and beluga spermatozoa (~40 % motility, evaluation under microscope).

To perform the multiple ICSI, diluted spermatozoa in PBS (0.001 %, 15°C) was filled in a microneedle and was not floated with the eggs in a petri dish. Each egg was microinjected with a single manipulation in the animal pole (~ 0.23 μl/egg). This corresponds to ~ 3827 sterlet spermatozoa per Siberian or Russian sturgeon egg and ~ 3905 Siberian sturgeon spermatozoa/beluga egg. Multiple ICSI were performed between Siberian sturgeon eggs and sterlet spermatozoa, between Russian sturgeon eggs and sterlet spermatozoa, as well as between beluga eggs and Siberian sturgeon spermatozoa.

All ICSI experiments were performed under a stereomicroscope (Leica M165 FC.) using a hydraulic injector (Cell-Tram Oil; Eppendorf, Germany) connected to a micromanipulator (MO-152; Narishige, Japan) on the animal pole of the eggs where the multiple micropyles are located. Application of a shallow injection was used during the ICSI technology, as it has been demonstrated in sturgeon microinjection techniques to result in high developmental rates (39). All transplants were incubated in PBS (15°C) for 30–40 min, washed three times with PBS to remove the floating spermatozoa in the petri dish, and then activated with filtrated water (15°C).

At the same time, *in vitro* fertilization of the control groups with fresh-stripped sperm was performed: beluga eggs x Siberian sturgeon spermatozoa (*n* = 31, fertilization rate = 90.3%), Siberian sturgeon eggs x sterlet spermatozoa (*n* = 80, fertilization rate = 90%), and Russian sturgeon eggs x sterlet spermatozoa (*n* = 87, fertilization rate = 90.8%) (Table 1). Moreover, *in vitro* fertilization of the control group with cryopreserved spermatozoa was performed: sterlet eggs x beluga spermatozoa (*n* = 583, fertilization rate = 51.5%) (Table 2). To confirm that spermatozoa were not motile nor sticky in the surface of eggs and had no ability to fertilize the eggs in the injection medium (PBS), we made a negative control group. We placed beluga eggs (*n* = 184) with Siberian sturgeon spermatozoa in PBS for 30 min, then we washed the eggs three times with PBS to remove the spermatozoa from the petri dish, and placed the eggs in filtrated water. Using the IBM SPSS statistic 27 software, Fisher's exact test was used to determine if there was a significant association in developing rate between the control group and ICSI experiments for each species gamete combination and developmental stage (Supplementary Figure S1). The negative control group exhibited neither fertilization nor embryonic development (Table 1).

**Table 1:** Developing rates of transplants after the different ICSI experiments using fresh-stripped spermatozoa and non-activated eggs.

**Experimental group**	**Experiments**	**Total eggs**	**Blastula (%)**	**Gastrula (%)**		**Neurula, hatching, feeding (%)**
				**Blastopore formation**	**2/3 Epiboly**	
Beluga egg-donor x	Pre-activated spermatozoon	26	22 (84.6)^**^	9 (34.6)^*^	4 (15.4)^*^	4 (15.4)^*^
Siberian sturgeon sperm-donor	Non-activated spermatozoon	39	23 (59.0)^**^	7 (17.9)^*^	6 (15.4)^*^	6 (15.4)^*^
	Multiple spermatozoa	63	40 (63.5)^**^	4 (6.3)	3 (4.8)	1 (1.6)
	Control	31	28 (90.3)	23 (74.2)	21 (67.7)	21 (67.7)
	Negative control	184	0 (0.0)	0 (0.0)	0 (0.0)	0 (0.0)
Siberian sturgeon egg-donor x	Pre-activated spermatozoon	90	73 (81.1)^**^	9 (10.0)	0 (0.0)	0 (0.0)
Sterlet sperm-donor	Non-activated spermatozoon	52	30 (57.7)^**^	22 (42.3)^**^	2 (3.8)	2 (3.8)
	Multiple spermatozoa	52	14 (26.9)^*^	5 (9.6)	0 (0.0)	0 (0.0)
	Control	80	72 (90.0)	63 (78.8)	59 (73.8)	59 (73.8)
Russian sturgeon egg-donor x	Pre-activated spermatozoon	54	44 (81.2)^**^	2 (4.5)	0 (0.0)	0 (0.0)
Sterlet sperm-donor	Non-activated spermatozoon	32	20 (62.5)^*^	2 (6.3)	2 (6.3)	2 (6.3)
	Multiple spermatozoa	62	16 (25.8)	14 (22.6)^**^	6 (9.7)	0 (0.0)
	Control	87	79 (90.8)	65 (74.7)	65 (74.7)	65 (74.7)

Negative control group (neither ICSI nor *in vitro* fertilization), and control groups (*in vitro* fertilization) at each developmental stage. Fisher's exact tests found statistically significant associations between the control and ICSI experiments at

* p < 0.05

or

** p < 0.001.

**Table 2:** Information of transplants after ICSI experiments with fresh-stripped spermatozoa used for the molecular analysis.

**ICSI Transplants**		
**Number**	**Identity-experiment**	**Developmental stage**
1	Pre-activated Siberian sturgeon spermatozoon x beluga egg	Gastrula (blastopore)
2	Pre-activated Siberian sturgeon spermatozoon x beluga egg	Larva 25dpa
3	Pre-activated Siberian spermatozoon x beluga egg	Larva 25dpa
4	Multiple Siberian sturgeon spermatozoa x beluga egg	Larva 25dpa
5	Non-activated Siberian sturgeon spermatozoon x beluga egg	Larva 25dpa
6	Non-activated Siberian sturgeon spermatozoon x beluga egg	Larva 25dpa
7	Non-activated Siberian sturgeon spermatozoon x beluga egg	Larva 25dpa
8	Non-activated Siberian sturgeon spermatozoon x beluga egg	Larva 25dpa
9	Non-activated Siberian sturgeon spermatozoon x beluga egg	Larva 25dpa
10	Pre-activated Siberian sturgeon spermatozoon x beluga egg	Larva 25dpa
11	Non-activated Siberian sturgeon spermatozoon x beluga egg	Larva 25dpa
12	Pre-activated Siberian sturgeon spermatozoon x beluga egg	Larva 25dpa
13	Multiple Siberian sturgeon spermatozoa x beluga egg	Gastrula (epiboly)
14	Multiple Siberian sturgeon spermatozoa x beluga egg	Gastrula (epiboly)
15	Non-activated sterlet spermatozoon x Siberian sturgeon egg	Larva 25dpa
16	Non-activated sterlet spermatozoon x Siberian sturgeon egg	Larva 25dpa
17	Non-activated sterlet spermatozoon x Russian sturgeon egg	Larva 25dpa
18	Non-activated sterlet spermatozoon x Russian sturgeon egg	Larva 25dpa
19	Multiple sterlet spermatozoa x Russian sturgeon egg	Gastrula (epiboly)
20	Multiple sterlet spermatozoa x Russian sturgeon egg	Gastrula (epiboly)
21	Multiple sterlet spermatozoa x Russian sturgeon egg	Gastrula (epiboly)
22	Multiple sterlet spermatozoa x Russian sturgeon egg	Gastrula (epiboly)
23	Multiple sterlet spermatozoa x Russian sturgeon egg	Gastrula (epiboly)
24	Multiple sterlet spermatozoa x Russian sturgeon egg	Gastrula (epiboly)

The numbers correspond to the identity of every transplant associated with the respective experiment, as well as its developmental stage.

Immediately after activation of the ICSI transplants and control groups with filtrated water, all embryos were treated with 0.01% tannic acid (SIGMA-ALDRICH^®^) alternated with filtrated water for 10 min to remove the egg surface stickiness. Development was observed at the two-cell stage, corresponding to 3–4 h post activation (hpa). At 10 hpa, forceps were used to remove the outer layers of chorion for better observation of the development. Developing embryos were placed in 0.01% penicillin and 0.01% streptomycin in filtered water (15°C) for 3 days. The inner layer of the chorion was removed at 5–6 days post activation. First feeding started after yolk resorption, 25 dpa. The freshwater annelid worm *tubifex sp*. was given twice per day at 8:00 am and 16:00 pm. Tanks were cleaned twice per day 2 h after the meal. All embryos and larvae were held at the ambient photoperiod at a water temperature of 15°C.

### 2.5. Molecular genotyping

Genomic DNA was extracted from caudal fin tissue of five egg-donors (a beluga sturgeon, a Siberian sturgeon, a Russian sturgeon, and two sterlets), three sperm-donors (a Siberian sturgeon, a sterlet, and a beluga sturgeon), along with 37 ICSI transplants, and one *in vitro* produced control embryo using GenElute Mammalian Genomic DNA Miniprep Kit (SIGMA-ALDRICH^®^) according to the manufacturer's instructions. The presence of the beluga sturgeon's genome in the respective ICSI transplants was investigated using beluga specific primer pair *153_HHp* + *153_uni* (44) that amplifies 153 bp fragment of beluga nuclear DNA. The presence of sterlet's genome in the respective ICSI transplants was tested by sterlet specific primer pair *247_ARp* + *247_uni* (44), which amplifies 247 bp fragment from sterlet nuclear DNA. The presence of Siberian or Russian sturgeon's genome in the respective ICSI transplants was tested using specific primer pair 395_ABp + 395_uni (45), which amplifies 395 bp fragment from Siberian or Russian sturgeon's nuclear DNA. All reactions were performed according to (44, 45) in two independent replicates. Together with the species-specific primers from above, the genomic constitution of the ICSI transplants was estimated by parentage-like assignment using six microsatellite markers: AciG_35 (46), AfuG_135 (47), Aox_45 (48), Spl_101, Spl_163, and Spl_173 (49). Amplification and microsatellite fragment analysis were carried out according to the protocol described by Havelka et al. (50). Genotypes were scored in GENEIOUS 8.1.9, using Microsatellite Plugin 1.4.4.

## 3. Results

### 3.1. ICSI using fresh-stripped spermatozoa

ICSI of non-activated single spermatozoa from Siberian sturgeon into beluga unfertilized eggs reconstructed 39 transplants, from which 23 (59.0 %) exhibited initial cleavages and reached the blastula stage. Seven (17.9%) reached the gastrula stage from which 6 (15.4 %) fulfilled epiboly, neurulation, hatching, and feeding until 25 dpa, upon which they were sacrificed. Pre-activated and immobilized single spermatozoa from Siberian sturgeon were injected into beluga unfertilized eggs reconstructing 26 transplants, from which 22 (84.6 %) exhibited initial cleavages and reached the blastula stage. Nine transplants (34.6 %) formed the blastopore in the gastrula stage and 4 (15.4 %) fulfilled the epiboly, the neurula stage, hatched, and fed until 25 days post activation (dpa), upon which they were sacrificed. ICSI using multiple spermatozoa with a single manipulation from Siberian sturgeon into beluga eggs resulted in reconstruction of 63 transplants from which 40 (63.5 %) exhibited initial cleavage and reached the blastula stage. Four transplants (6.3 %) entered the gastrula stage, three (4.8 %) fulfilled epiboly, and one (1.6 %) entered the neurula stage, hatched, and fed until 25 dpa, upon which it was sacrificed (Table 1).

Sterlet pre-activated and immobilized single spermatozoa were injected into Siberian sturgeon unfertilized eggs reconstructing 90 transplants, from which 73 (81.1 %) exhibited initial cleavages and reached the blastula stage. Nine transplants (10.0 %) formed the blastopore at gastrula stage and stopped development. ICSI of non-activated single spermatozoa from sterlet into Siberian sturgeon unfertilized eggs reconstructed 52 transplants, from which 30 (57.7 %) exhibited initial cleavages and reached the blastula stage. Twenty-two (42.3 %) reached the gastrula stage from which 2 (3.8 %) fulfilled epiboly, entered the neurula stage, hatched, and fed until 25 dpa, upon which they were sacrificed. ICSI using multiple spermatozoa with a single manipulation from sterlet into Siberian sturgeon eggs resulted in reconstruction of 52 transplants from which 14 (26.9 %) exhibited initial cleavage and reached the blastula stage. Five (9.6 %) reached the gastrula stage at blastopore formation and stopped development (Table 1).

Sterlet pre-activated (15 s) and immobilized single spermatozoa were injected into Russian sturgeon unfertilized eggs reconstructing 54 transplants, from which 44 (81.2 %) exhibited initial cleavages and reached the blastula stage. Two transplants (4.5 %) formed the blastopore at gastrula stage and stopped development. ICSI of non-activated single spermatozoa from sterlet into Russian sturgeon unfertilized eggs reconstructed 32 transplants, from which 20 (62.5 %) exhibited initial cleavages and reached the blastula stage. Two (6.3 %) reached the gastrula stage, the neurula stage, hatched, and fed until 25 dpa, upon which they were sacrificed. ICSI using multiple spermatozoa with a single manipulation from sterlet into Russian sturgeon eggs resulted in reconstruction of 62 transplants from which 16 (25.8 %) exhibited initial cleavage and reached the blastula stage. Fourteen (22.6 %) reached the gastrula stage at blastopore formation and 6 (9.7 %) fulfilled epiboly and stopped development (Table 1).

### 3.2. Molecular analysis after ICSI using fresh-stripped spermatozoa

In total, 24 ICSI transplants were analyzed for the presence of DNA of both egg- and sperm-donor species (Table 2).

**Figure 1:**
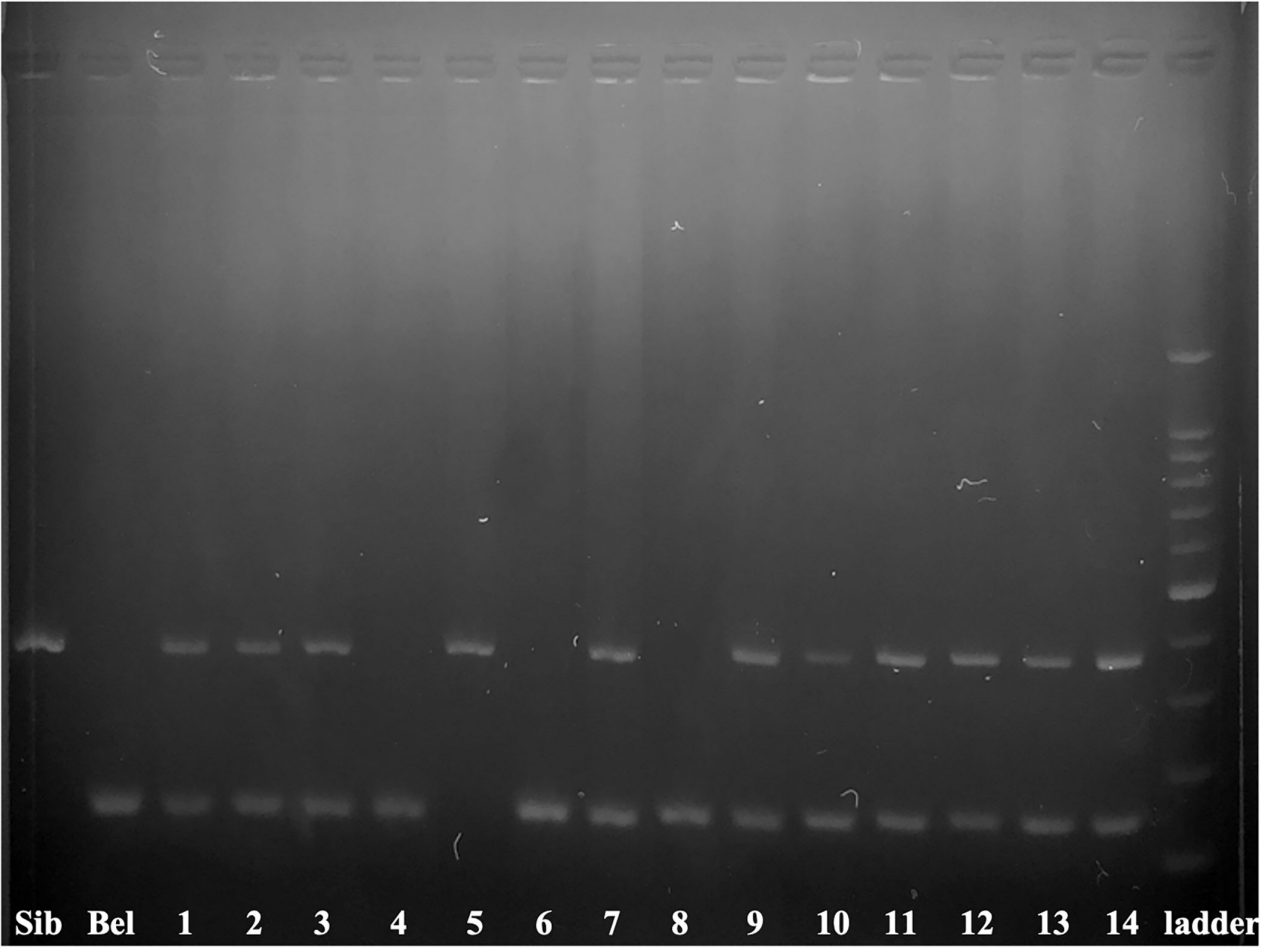
Electrophoretogram of 395_AB and 153_HH markers in 14 ICSI produced embryos. The marker 395_AB amplifies 395 bp band only when the sample contains Siberian and/or Russian sturgeon nuclear DNA while the marker 153_HH amplifies 153 bp band only when a sample contains nuclear DNA of beluga. Numbers correspond to the embryos and larvae of Table 2. Siberian sturgeon fin (Sib) and beluga fin (Bel) are used as positive controls 100–1500 bp ladder.

The contribution of Siberian sturgeon sperm-donor DNA in developing embryos was studied in 14 specimens (Figure 1). We combined two markers: 395_AB (45) and 153_HH (44). The marker 395_AB amplifies 395 bp band only if a sample contains Siberian's and/or Russian's sturgeon genomic DNA while the marker 153_HH amplifies 153 bp band only if a sample contains genomic DNA of beluga. Ten transplants coming from the group ICSI with pre-activated single spermatozoa (*n* = 5), from the group ICSI with non-activated single spermatozoa (*n* = 3), and from the group of ICSI with multiple spermatozoa (*n* = 2) showed presence of DNA from both parents, thus confirming successful incorporation of sperm-donor DNA in the transplants (71.4 %). One transplant from the group ICSI using non-activated single spermatozoa showed contribution of only sperm-donor's DNA without any contribution from the beluga egg-donor (7.1 %). Three transplants showed amplification of DNA only from females (21.4 %) from the group ICSI with multiple spermatozoa (*n* = 1) and from the group ICSI with non-activated spermatozoa (*n* = 2) (Figure 1).

**Figure 2:**
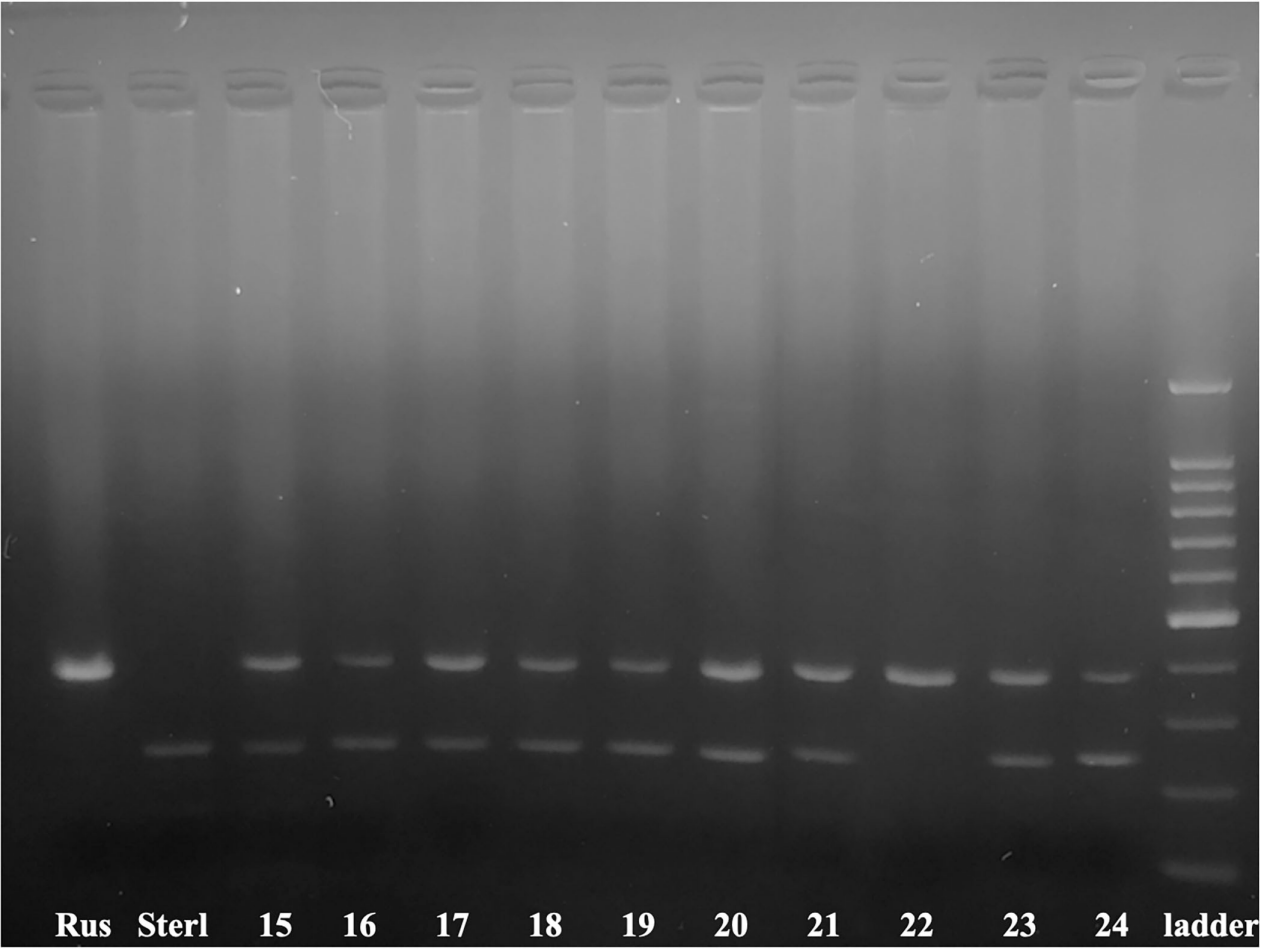
Electrophoretogram of 395_AB and 247_AR markers in 10 ICSI produced transplants. The marker 395_AB amplifies 395 bp band only when a sample contains Siberian and/or Russian sturgeon nuclear DNA and the marker 247_AR amplifies 247 bp band only when a sample contains nuclear DNA of sterlet. Numbers correspond to the embryos and larvae of Table 2. Russian sturgeon fin (Rus) and sterlet fin (Sterl) are positive controls 100–1500 bp ladder.

Efficiency of ICSI was studied in 10 transplants (Figure 2), by species specific markers. We combined two markers: 395_AB (45) and 247_AR (44). The marker 247_AR amplifies 247 bp band only if a sample contains genomic DNA of sterlet. Consequently, 9 specimens (90 %) showed presence of DNA from both parents, from the group ICSI using non-activated single sterlet spermatozoa into Siberian sturgeon's eggs (*n* = 2), the group ICSI using non-activated single sterlet spermatozoa into Russian sturgeon's eggs (*n* = 2), and the group ICSI using multiple sterlet spermatozoa into Russian sturgeon's eggs (*n* = 5). One specimen showed development of only egg-donor's DNA (10 %) in the group ICSI using multiple spermatozoa from sterlet into Russian sturgeon's eggs (Figure 2).

Microsatellite analysis of the ICSI transplants from Table 2 showed that 19 transplants (N°1–3, 7, 9–21, 23, and 24) contained both egg- and sperm-donor's DNA. One transplant (N°21) contained two haploid genomes from the sperm-donor according to the microsatellite markers *AfuG_135* and *Spl_163*. Furthermore, one transplant (N°5) contained only sperm-donor's DNA, without any genomic contribution from the egg-donor (Table 3).

**Table 3:** Molecular analysis with microsatellite markers after different ICSI experiments using fresh-stripped spermatozoa.

**N°**	**Markers**														
	***395_AB***	***247_AR***	***153_HH***	***AciG_35***		***AfuG_135***		***Aox_45***		***Spl_163***		***Spl_101***		***Spl_173***	
				**D**	**R**	**D**	**R**	**D**	**R**	**D**	**R**	**D**	**R**	**D**	**R**
1	+		+	+	+	+	+	+	+	+	+	N/A	N/A	N/A	N/A
2	+		+	+	N/A	+	+	+	+	+	+	N/A	N/A	N/A	N/A
3	+		+	+	N/A	+	+	+	+	+	+	N/A	N/A	N/A	N/A
4	–		+	N/A	+	–	+	–	+	–	+	N/A	N/A	N/A	N/A
5	+		–	+	N/A	+	–	+	–	+	–	N/A	N/A	N/A	N/A
6	–		+	N/A	N/A	–	+	–	+	–	+	N/A	N/A	N/A	N/A
7	+		+	N/A	N/A	+	+	+	+	+	+	N/A	N/A	N/A	N/A
8	–		+	N/A	+	–	+	–	+	–	+	N/A	N/A	N/A	N/A
9	+		+	+	+	+	+	+	+	+	+	N/A	N/A	N/A	N/A
10	+		+	+	N/A	+	+	+	+	+	+	N/A	N/A	N/A	N/A
11	+		+	+	+	+	+	+	+	+	+	N/A	N/A	N/A	N/A
12	+		+	+	N/A	+	+	+	+	+	+	N/A	N/A	N/A	N/A
13	+		+	+	N/A	+	+	+	+	+	+	N/A	N/A	N/A	N/A
14	+		+	+	+	+	+	+	+	+	+	N/A	N/A	N/A	N/A
15	+	+		+	+	+	+	+	+	N/A	N/A	+	+	+	+
16	+	+		N/A	+	+	+	+	+	N/A	N/A	+	+	+	+
17	+	+		N/A	+	+	+	+	+	+	+	+	+	N/A	N/A
18	+	+		+	+	+	+	N/A	+	+	+	N/A	+	N/A	N/A
19	+	+		+	+	+	+	+	+	+	+	+	+	N/A	N/A
20	+	+		+	+	+	+	+	+	+	+	N/A	+	N/A	N/A
21	+	+		N/A	N/A	+ (2x)	+	+	+	+ (2x)	+	N/A	+	N/A	N/A
22	+	–		N/A	+	–	+	–	+	–	+	N/A	+	N/A	N/A
23	+	+		+	+	+	+	+	+	+	+	+	+	N/A	N/A
24	+	+		N/A	+	+	+	+	+	+	+	+	+	N/A	N/A

Numbers correspond to the embryos and larvae of Table 2. The marker 395_AB is a specific marker of Siberian and Russian sturgeon. The marker 247_AR is a specific marker for sterlet and the marker 153_HH is a specific marker for beluga. D = sperm-donor; R = egg-donor; + = informative allele(s) present in allele phenotype of the sample; – = informative allele(s) not present in allele phenotype of the sample; N/A = not informative locus.

### 3.3. ICSI using cryopreserved spermatozoa

Non-activated single cryopreserved spermatozoa from beluga sturgeon (~ 40 % motility) were injected into sterlet unfertilized and non-activated eggs. Ninety-seven transplants reconstructed, from which 22 (22.7 %) exhibited initial cleavages and reached the blastula stage. Thirteen transplants (9.3 %) formed the blastopore, 10 (6.2 %) fulfilled the epiboly, and 4 (4.1 %) reached the neurula stage (Table 4).

**Table 4:** Developing rates of transplants after ICSI using single non-activated cryopreserved beluga spermatozoa (~ 40 % motility) into non-activated sterlet eggs and control group at each developmental stage.

**Experimental group**	**Total eggs**	**Blastula (%)**	**Gastrula (%)**		**Neurula (%)**
			**Blastopore formation**	**2/3 Epiboly**	
Non-activated spermatozoa	97	22 (22.7)	13 (9.3)	10 (6.2)	4 (4.1)
Control	583	300 (51.5)	224 (38.4)	224 (38.4)	224 (38.4)

### 3.4. Molecular analysis after ICSI using cryopreserved spermatozoa

In total, 13 ICSI transplants were analyzed for the presence of DNA of both egg- and sperm-donor species (numbers 25–33 and 35–38) and one control embryo (number 34) that was produced after *in vitro* fertilization of beluga cryopreserved spermatozoa to sterlet eggs. The transplants were created after ICSI using single cryopreserved spermatozoa from beluga into sterlet eggs. Numbers 25–27 arrested at gastrula stage in blastopore formation, 28–33 arrested at gastrula stage at 2/3 epiboly, and 35–38 arrested at neurula stage. The control embryo (number 34) arrested at neurula stage (Table 5).

**Table 5:** Information of transplants after ICSI with single cryopreserved beluga spermatozoa into sterlet eggs and one *in vitro* produced control embryo used for the molecular analysis.

**Information of embryos**		
**N** ^o^	**Identity**	**Developmental stage**
25	ICSI transplant	Gastrula (blastopore)
26	ICSI transplant	Gastrula (blastopore)
27	ICSI transplant	Gastrula (blastopore)
28	ICSI transplant	Gastrula (2/3 epiboly)
29	ICSI transplant	Gastrula (2/3 epiboly)
30	ICSI transplant	Gastrula (2/3 epiboly)
31	ICSI transplant	Gastrula (2/3 epiboly)
32	ICSI transplant	Gastrula (2/3 epiboly)
33	ICSI transplant	Gastrula (2/3 epiboly)
34	*In vitro* fertilized control	Neurula
35	ICSI transplant	Neurula
36	ICSI transplant	Neurula
37	ICSI transplant	Neurula
38	ICSI transplant	Neurula

The numbers correspond to the identity of every embryo, as well as its developmental stage.

**Figure 3:**
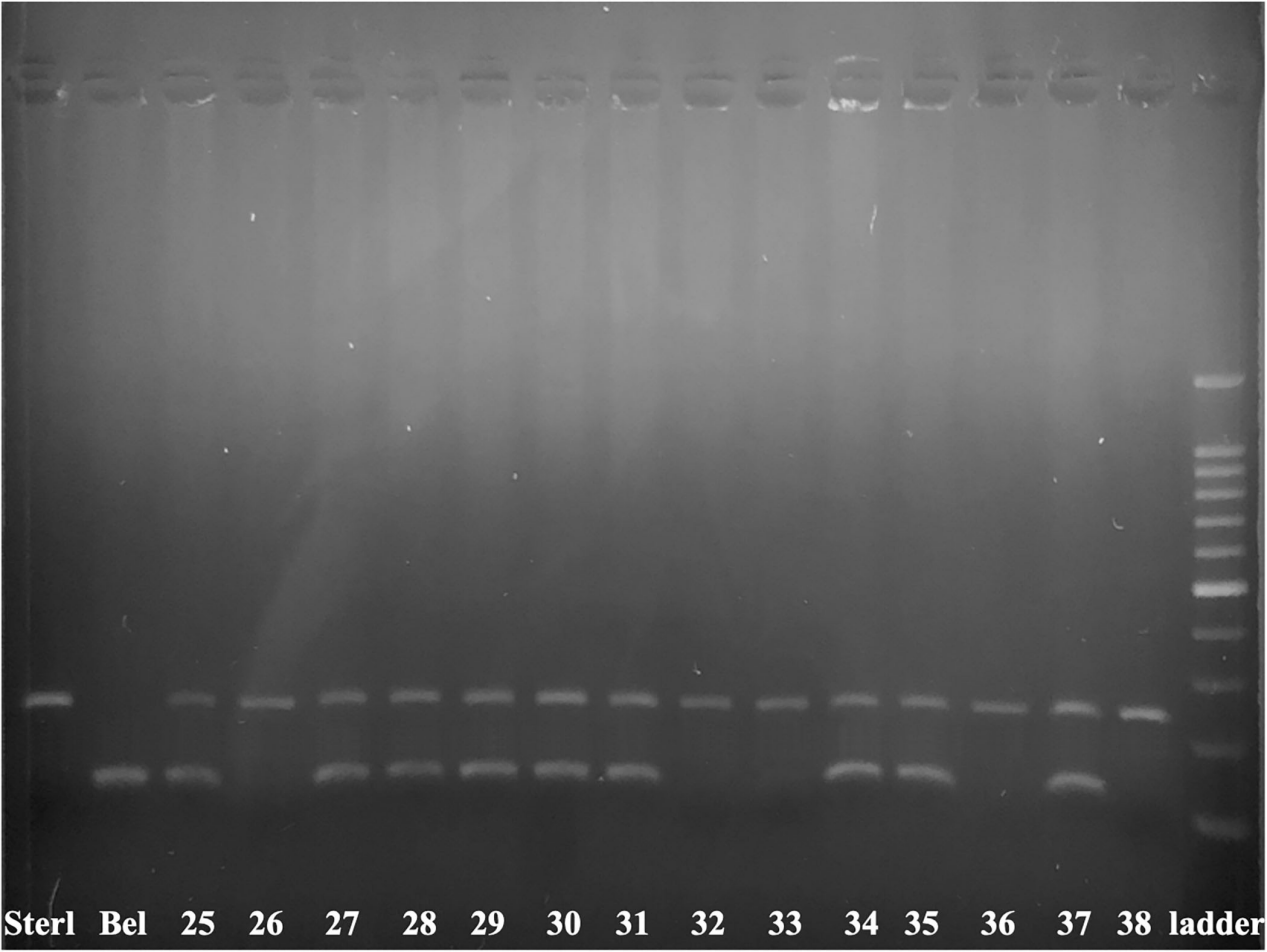
Electrophoretogram of 153_HH and 247_AR markers in 14 produced embryos. The marker 247_AR amplifies 247 bp band only when the sample contains nuclear DNA of sterlet, while the marker 153_HH amplifies the 153 bp band only when the sample contains nuclear DNA of beluga. Sterlet fin (Sterl) and beluga fin (Bel) are positive controls. The numbers 25–33, 35–38 are corresponding to transplants after ICSI of single non-activated cryopreserved beluga spermatozoa into sterlet's eggs. Number 34 is a control embryo produced after *in vitro* fertilization of cryopreserved beluga spermatozoa with sterlet eggs 100–1500 bp ladder.

Molecular analysis of 14 embryos has been performed by species specific markers (Figure 3, Table 6). We combined two markers: *247_AR* and *153_HH* (44). Eight ICSI transplants (61.5 %) showed presence of DNA from both parents, while 5 (38.5 %) showed development of only sterlet egg-donor's DNA without contribution of beluga sperm-donor's DNA (Figure 3, Table 6).

**Table 6:** Molecular analysis with species specific markers after ICSI using single cryopreserved spermatozoa from beluga sturgeon into sterlet eggs.

**N^o^**	**Markers**	
	***153_HH***	***247_AR***
25	+	+
26	–	+
27	+	+
28	+	+
29	+	+
30	+	+
31	+	+
32	–	+
33	–	+
34	+	+
35	+	+
36	–	+
37	+	+
38	–	+

Numbers correspond to the embryos from Table 5. The marker 153_HH is a specific marker for beluga while the 247_AR is a specific marker for sterlet.

### 3.5. Phenotypic analysis of transplants after the ICSI technology

All resultant embryos and larvae followed a normal developmental pattern (38, 39, 51) without visible abnormalities in shape.

## 4. Discussion

As far as we know, this is the first study of ICSI technology in sturgeon, the most critically endangered fish in the world (37). In this study, different egg-sperm species combinations were used, ranging between endangered to critically endangered. We chose to perform ICSI using different species because sturgeon hybridize naturally, producing viable fertile offspring (52–55), and are used widely for aquaculture purposes (54).

ICSI technology does not require motile spermatozoa to produce healthy offspring as long as the sperm nucleus has intact genetic integrity (56, 57). Egg activation (7, 34, 35) or non-activation (19, 34) have been used in fish ICSI. After sturgeon ICSI, feeding larvae were produced in most experimental groups when fresh-stripped sperm injected (up to 15.4 %). Comparing our results with ICSI in teleost fish species which exhibit meroblastic embryonic development, various hatching rates are shown: 1.6 % in zebrafish (34), 8.5 % in Nile tilapia (19), and 13.4 % (7) and 6.7 % (36) in medaka. In amphibians which exhibit holoblastic embryonic development similar to sturgeon embryos (51), after ICSI of African clawed frog the success rate of production metamorphosed larvae was 0.7 % (58).

All the experimental groups with the fresh-stripped spermatozoa (ICSI of single pre-activated or non-activated spermatozoa and ICSI of multiple spermatozoa) and the cryopreserved spermatozoa group (ICSI of single non-activated spermatozoa) exhibited embryonic development. The critical stage proved to be the blastopore formation in gastrula stage, suspected as the stage in which the embryonic genes are expressing in sturgeon (59). The inability of the embryos to surpass that stage is similar to the results after sturgeon SCNT (38, 39). Comparing the embryonic developmental success after ICSI of single fresh-stripped spermatozoa to SCNT using single fin-cells (38), we observed an 8.6- to 12.6-fold higher success of development (blastula stage). Success in human ICSI compared to human SCNT has been attributed to fewer acquired mutations in spermatozoa compared to somatic cells (5- to 10-fold) from the same individual (60). When comparing the ICSI using multiple spermatozoa (~4000/egg) with the SCNT using multiple fin-cells (~300/egg) (39), we observed the same or half percentage of developing blastula embryos. This may be attributed to the nearly 13-fold higher cells transplanted in the case of ICSI using multiple spermatozoa compared to the SCNT using multiple fin-cells.

In all egg-sperm species-combination groups the experiment of ICSI using single fresh-stripped non-activated spermatozoa produced normal-shaped feeding larvae. In human ICSI, it is common practice to use pre-activated spermatozoa (61). In the present study, the experiment of ICSI using single fresh-stripped pre-activated spermatozoa produced normal-shaped feeding larvae only in one group. On the contrary, Psenicka et al. (40) supported that activation of acrosomal reaction in sturgeon spermatozoa (activated spermatozoa) makes them more successful in *in vitro* fertilization and transplants' hatching rate. After ICSI of multiple fresh-stripped spermatozoa, all three egg-sperm species-combination groups were able to form the blastopore in gastrula stage while only one group produced larvae. In that group, all experiments of ICSI were successful (production of feeding larvae), with single spermatozoa transplantation (15.4 % in both experiments; pre-activated and non-activated) being much more successful than the multiple spermatozoa transplantation (1.6 %). On the contrary, after sturgeon SCNT the use of multiple fin cells proved to be more promising with high rates of embryonic development (39). This probably happened because injection of ~4000 spermatozoa compared to ~300 fin cells creates significant pressure for the recipient egg. The sturgeon egg must have a saturation point in its ability to digest such a large number of spermatozoa permitting embryonic development.

Ciereszko et al. (62) suggested that cryopreserved spermatozoa will become a standard practice in future reproduction in sturgeon fisheries. Indeed, fertility preservation strategies using cryopreservation have enormous potential for helping sustain and protect rare and endangered species. Gametes' cryopreservation could help to maintain the species genetic heterozygosity, while minimizing movement of living animals (63). However, cryopreservation of sturgeon spermatozoa affects their ability to fertilize, due to a decrease of sperm motility function and induction of acrosomal damage (64, 65). In this study, single non-activated cryopreserved beluga spermatozoa fertilized sterlet eggs *in vitro* (control group) and after ICSI showed embryonic development (blastula stage) of 51.5 and 22.7 %, respectively. Some embryos from the ICSI group were able to reach the neurula stage (4.1 %) and stopped development. Studies of Nile tilapia ICSI using cryopreserved spermatozoa produced embryos until blastula stage (20 %) (19). The authors suggested that the spermatozoa selected for injections were damaged, compromising further development, something that probably happened in our study. Ice crystal formation due to cryopreservation can disrupt the sperm membrane, resulting in loss of cytoplasmic contents that may contribute to egg activation. Ice crystals can also damage the chromatin, resulting in fragmented DNA. Injection of eggs with spermatozoa containing fragmented chromatin has resulted in failure of spermatozoa decondensation, fertilization, and embryonic development in humans (66, 67).

After the different experiments of sturgeon ICSI we did not observe any developmental malformation in the resulting embryos and larvae. Sturgeon species are proving very good model species and could be used extensively for embryonic experimental studies. Fail of teleost ICSI to produce normal-shaped embryos and larvae could be attributed to the difficulty to inject through the micropyle of eggs. Precise control of the injection site of spermatozoon to the egg seems to be a prerequisite for successful medaka ICSI (7). In the case of zebrafish ICSI, stress concentration was observed in the contact area between the injection micropipette and the zebrafish egg, which causes egg breakage resulting in embryonic malformation and death (68).

Molecular analysis revealed that most of the ICSI transplants exhibit the genome of both egg- and sperm-donor species. This result confirms that sturgeon ICSI can be used as an assisted reproductive technology for producing offspring of selective genitors. Application of ICSI among sturgeons could potentially benefit the breeding facilities for consuming purposes. On the other hand, we observed ICSI transplants with only egg-donor or only sperm-donor DNA. We have already observed transplants after SCNT using a single or multiple fin-cells that expressed only egg-donor's DNA due to unusual disruption in early embryogenesis (38, 39). In the case of only sperm-donor-derived ICSI transplants we believe that the egg during the microinjections was exposed to a massive stress. Rouillon et al. (69) found that after goldfish SCNT the production of only sperm-donor-derived offspring happened due to an alteration of the meiotic furrow and the egg-donor DNA not contributing in the development. It is possible that the same happened in the present study. In the future it will be useful to perform ploidy analysis of the resulted embryos and larvae.

Our study clearly demonstrates that the ICSI technology is feasible for sturgeon's reproduction, but further studies are needed to improve efficiency. The development of ICSI technology in sturgeon could yield valuable applications. The sturgeon caviar industry is quite demanding and sex pre-selection of offspring through the use of sexed spermatozoa has great potential, because the production of only female sturgeons can result in higher production of caviar. Furthermore, producing predominantly female offspring is advantageous in accelerating the repopulation rate, especially in species that are notorious for slow reproduction (70), like in the case of sturgeon. The positive results shown here demonstrate that ICSI could be used as another genetic tool for improvement in aquaculture. ICSI would be best suited to reconstitution of desired lines from selected genitors and therefore using a small brood stock to produce large numbers of sturgeon with preferable characteristics.

## Data availability statement

The original contributions presented in the study are included in the article/Supplementary material, further inquiries can be directed to the corresponding author.

## Ethics statement

The animal study was reviewed and approved by Animal Research Committee of the University of South Bohemia in Ceské Budějovice (Reference Number: 2293/2015-MZE-17214).

## Author contributions

EF designed, conducted the ICSI experiments, analyzed data, and wrote the manuscript. MH performed and analyzed the molecular analysis. MP managed the laboratory and provided necessary suggestions on the text together with TS. JL performed the statistical analysis and data visualization. MR and DG handled the sturgeon spawning. All authors contributed to the preparation of the manuscript and approved the final version.
