# Supplementary material for: Intracytoplasmic sperm injection in sturgeon species: A promising reproductive technology of selected genitors
Source: Front Vet Sci. 2022 Dec 23;9:1054345. doi: 10.3389/fvets.2022.1054345 (PMC9816131; doi:10.3389/fvets.2022.1054345)
Supplement: Supplementary file 1 [file Image_1.pdf]

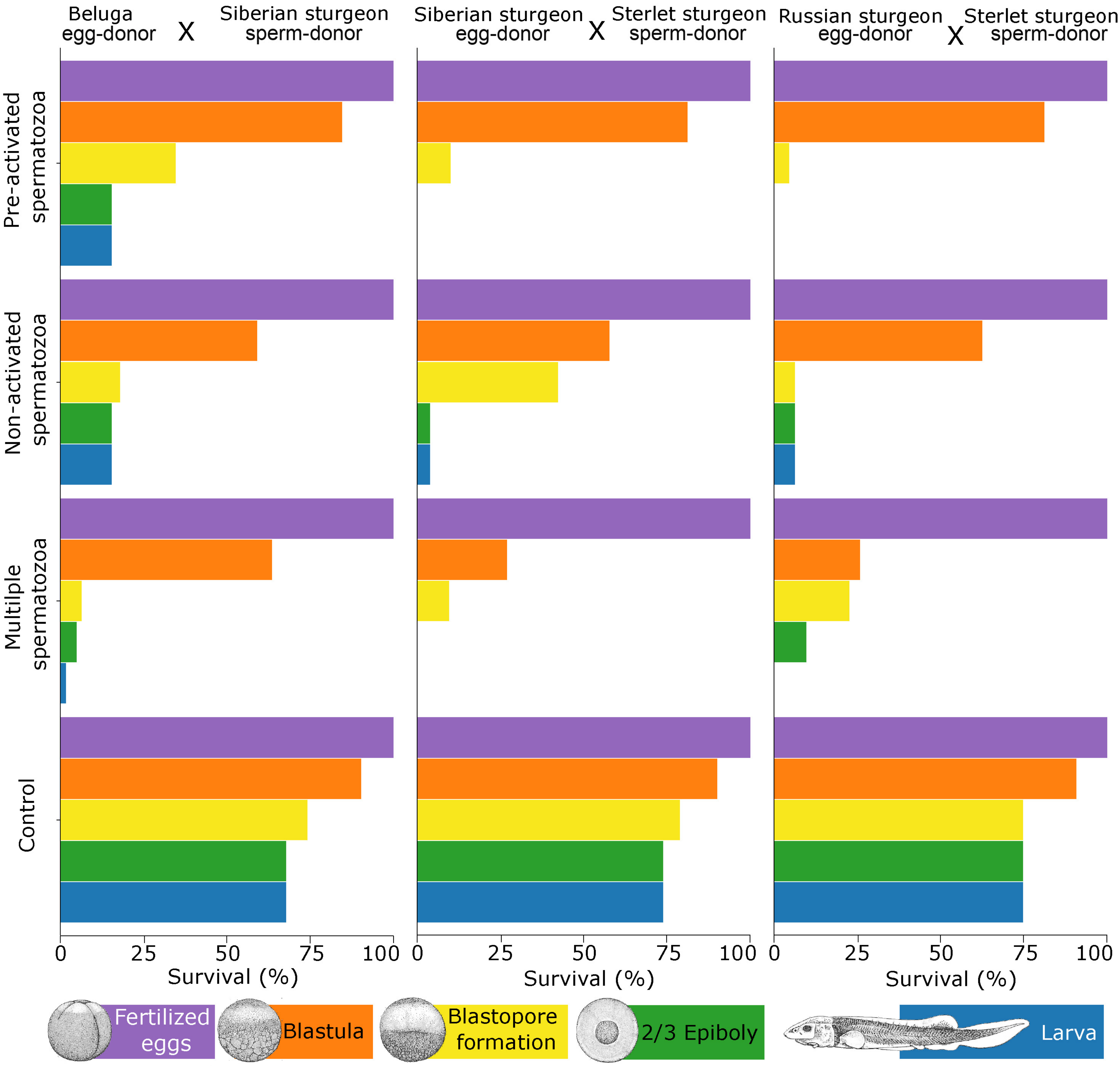

Supplementary Figure 1. Survival rates of transplants at each developmental stage after the different ICSI experiments using fresh-stripped spermatozoa and non-activated eggs. For control groups *in vitro* fertilization was performed.
